# Menstrual characteristics: Undervalued risk factors for adverse pregnancy outcomes

**DOI:** 10.1111/ppe.13136

**Published:** 2024-11-11

**Authors:** Yihui Yang, Donghao Lu

**Affiliations:** ^1^ Unit of Integrative Epidemiology Institute of Environmental Medicine, Karolinska Institutet Stockholm Sweden

Adverse pregnancy outcomes (APO), such as gestational diabetes mellitus (GDM), hypertensive disorders of pregnancy, and preterm delivery, are significant health challenges among women during the perinatal period. In the United States, 20% of pregnancies are affected by at least one APO.^1^ Studies have shown that APOs can contribute to women's lifelong health^1^; hence, it remains crucial to identify risk factors of APOs for early screening and intervention in high‐risk populations.

For decades, attempts have been made to study the risk factors of APOs, most of which were identified during or close to the perinatal window, for example, premorbid diseases and tobacco use during pregnancy.^2^ Notably, although menstrual health is believed to be vital to women's well‐being, it is often neglected in perinatal care, given the complete absence of menstruation during pregnancy. However, emerging evidence has shown that the timing of menarche is associated with a higher risk of several APOs, including GDM,^3^ hypertensive disorders of pregnancy^4^ and preterm delivery,^5^ suggesting that menstrual characteristics may play a role in women's perinatal health.

In this issue of *Paediatric and Perinatal Epidemiology*, Wang and colleagues^6^ confirmed previous findings that women with early menarche were at a higher risk of GDM. Based on data from 30,473 participants in a US‐based digital Apple cohort, they found early menarche was consistently associated with three GDM outcomes, including GDM in the first pregnancy (risk ratio [RR] 1.34, 95% confidence interval [CI] 1.15, 1.57), across pregnancies (RR 1.24, 95% CI 1.10, 1.39) and recurrent GDM (odds ratio [OR] 1.51, 95% CI 1.21, 1.89). The robustness of their findings was reaffirmed in several sensitivity analyses, including using inverse probability weights to account for selection bias due to non‐response. These findings suggest that for women with early menarche, clinicians may be aware of the higher risk of GDM in each of their pregnancies.

In addition to the timing of menarche, other menstrual characteristics associated with APOs are less investigated. The study by Wang et al.^6^ found an association between prolonged time‐to‐regularity and the risk of GDM. Although previous studies found irregular menstrual cycles in mid‐adulthood were associated with GDM,^7^ Wang et al. first illustrated prolonged time‐to‐regularity in early life was associated with GDM in the first pregnancy (RR 1.22, 95% CI 1.08, 1.38), across pregnancies (RR 1.16, 95% CI 1.05, 1.27) and recurrent GDM (OR 1.19, 95% CI 0.99, 1.43). While these findings need to be replicated in other populations, factoring in various aspects of menstrual characteristics might help better inform GDM risk identification.

While these findings are provocative, some caution should be exercised regarding clinical implications. Most studies were based on cross‐sectional or retrospective designs,^5^, ^8^ in which exposures and outcomes were surveyed simultaneously. For example, in the study by Wang et al.,^6^ it is not implausible that women who have GDM recalled their menstrual health differently compared to those without GDM. This may have contributed to differential misclassification, potentially biasing their findings towards an unknown direction.

In addition, most studies utilised secondary data and, therefore, may not collect information on important confounders specific to the research question. In the study by Wang et al.,^6^ the authors have adjusted for a range of potential confounders and even a family history of diabetes and other metabolic conditions. However, childhood abuse, which is associated with both early menarche^9^ and GDM,^10^ remains unaccounted for, suggesting the possibility of unmeasured confounding bias.

We propose several future research directions to better understand the relationship between menstrual characteristics and APOs.

1. Broaden definitions of menstrual characteristics. The timing of menarche is the most commonly studied exposure when relating menstrual characteristics to APOs. However, studies may explore other menstrual characteristics in post‐menarche years as potential risk factors of APOs, such as menstrual cycle length and intensity of menstrual flow, to identify additional menstruation‐related risk factors for APOs.
2. Assess APOs comprehensively. Previous research mainly focused on common pregnancy complications and birth outcomes. However, a more comprehensive assessment of pregnancy outcomes, expanding to other less common pregnancy adversities and maternal mental health, can better gauge the impact of menstrual characteristics on perinatal health holistically.
3. Collect data prospectively. Prospective cohorts that start follow‐up from early life or at least before pregnancy are still needed. Such cohorts are important in minimising misclassification bias of exposures, outcomes and confounders. In addition, ongoing longitudinal studies may consider using menstrual tracking apps to collect dynamic data on menstrual characteristics.
4. Understand the mediators/pathways. The mechanisms between early‐life menstrual characteristics and APOs are still unknown. Future studies may explore the role of potential mediators, including physical activity, diet and hormones, to advance our understanding of the mechanisms underlying APOs and informing prevention strategies for women who have adverse menstrual characteristics in their early lives.
5. Incorporate menstrual characteristics in predicting APOs. Future studies may evaluate the efficacy of menstrual characteristics in predicting APOs and investigate whether combinations of menstrual characteristics with other well‐established risk factors have better predictive values for APOs.

The paper by Wang et al. provides important insights into our understanding of the association between early‐life menstrual characteristics and the risk of APOs. Although the current evidence may not be adequate to support the causation, these findings, together with previous studies highlight the importance of factoring in menstrual characteristics for risk stratification and early detection of APOs in perinatal care. We hope future studies will continue to bring further understanding to this important but understudied topic, making it possible to assess the risk of APOs before or in early pregnancy.

## ABOUT THE AUTHORS

**Yihui Yang** is a PhD candidate at Karolinska Institutet, Stockholm, Sweden. She studies women's mental health using an epidemiological approach. Specifically, she studies premenstrual disorders as well as sex differences in psychiatric disorders, using data from international population‐based cohorts.

**Donghao Lu** is an Associate Professor (Docent) in Epidemiology and Research Group Leader of the Women's Mental Health Epidemiology Group at Karolinska Institutet, Stockholm, Sweden. His research is focused on women's mental health over the life course to understand the underlying mechanisms affecting women's mental health and potential health consequences.

## AUTHOR CONTRIBUTIONS

YY drafted the commentary, and DL critically revised and reviewed it.

## CONFLICT OF INTEREST STATEMENT

None.
